# The impact of attending day care designed for home-dwelling people with dementia on nursing home admission: a 24-month controlled study

**DOI:** 10.1186/s12913-018-3686-5

**Published:** 2018-11-16

**Authors:** Anne Marie Mork Rokstad, Knut Engedal, Øyvind Kirkevold, Jūratė Šaltytė Benth, Geir Selbæk

**Affiliations:** 1 Norwegian National Advisory Unit on Ageing and Health, Vestfold Hospital Trust, Postbox 2136, 3103 Tønsberg, Norway; 2 Faculty of Health Sciences and Social care, Molde University College, Molde, Norway; 3 Department of Care and Nursing, Faculty of Health, Norwegian University of Science and Technology (NTNU), Gjøvik, Norway; 4 The Research Centre for Age-Related Functional Decline and Disease, Innlandet Hospital Trust, Ottestad, Norway; 5 Institute of Clinical Medicine, Campus Ahus, University of Oslo, Oslo, Norway; 6 Health Services Research Unit, Research Centre, Akershus University Hospital, Postbox 1000, Lørenskog, 1478 Oslo, Norway; 7 Department of Nursing Science, Faculty of Medicine, University of Oslo, Oslo, Norway

**Keywords:** Dementia, Day care, Nursing home admission

## Abstract

**Background:**

Day care services offer meaningful activities, a safe environment for attendees and respite for family caregivers while being expected to delay the need for nursing home (NH) admission. However, previous research has shown inconsistent results regarding postponement of NH admission. The objective of the study was to explore the influence of a day care programme designed for home-dwelling people with dementia on NH admission.

**Method:**

A quasi-experimental trial explored the proportion of patients permanently admitted to nursing homes after 24 months as the main outcome by comparing a group of day care attendees (DG) and a group of participants without day care (CG). In all, 257 participants were included (181 in DG and 76 in CG). A logistic regression model was developed with NH admission as the outcome. Participant group (DG or CG) was the main predictor, baseline patient and family caregiver characteristics and interactions were used as covariates.

**Results:**

The mean age of participants was 81.5 (SD 6.4), 65% were women and 53% lived alone. The mean MMSE score was 20.4 (SD 3.5). In all, 128 (50%) of the participants were admitted to a nursing home by the 24-month follow-up, 63 participants (25%) completed the follow-up assessment and 66 (26%) dropped out due to death (8%) and other reasons (18%). In the logistic unadjusted regression model for NH admission after 24 months, participant group (DG or CG) was not found to be a significant predictor of NH admission. The results from the adjusted model revealed that the participant group was associated with NH admission through the interactions with age, living conditions, affective symptoms, sleep symptoms and practical functioning, showing a higher probability for NH admission in DG compared to CG.

**Conclusion:**

The study reveals no evidence to confirm that day care services designed for people with dementia postpone the need for NH admission. Admission to nursing homes seems to be based on a complex mix of personal and functional characteristics both in the person with dementia and the family caregivers. The findings should be considered in accordance with the limitation of inadequate power and the high drop-out rate.

**Trial registration:**

The study is registered in Clinical Trials (NCT01943071).

## Background

Day care services for people with dementia aim to offer meaningful activities and a safe environment to improve the quality of life of the attendees. Additionally, these services should act as a respite service for family caregivers, and it is suggested that such services could delay the need for nursing home (NH) admission [1–5].

In Norway, day care centre programmes, as well as domiciliary nursing and nursing home care, are provided by the local authorities. The municipalities have the responsibility for offering good quality day care services to older people, including people with dementia [6]. The provision of day care services specially designed for people with dementia was one of the main priorities of the first governmental Norwegian Dementia Plan [7], and this commitment has been maintained in the second Dementia Plan [8]. Local authorities in Norway are offered funding from the government to provide day care services for this group of patients close to their own homes. One of the reasons why the Ministry of Health and Social Care is prioritising this is that a day care programme is less expensive than care in nursing homes or other living accommodations [9]. A repeated national survey investigating the municipal dementia care services in Norway revealed that the percentage of municipalities offering day care for people with dementia increased from 30% in 2007 to 71% in 2014 [10].

Dementia is the main predictor of NH admission among Norwegian community-dwelling people 70 years and older who receive home care services [11]. Predictors of NH admission for people with dementia have been reported in several systematic reviews. Advanced age has been found to increase the probability of NH admission in dementia patients [12, 13]. The meta-analysis of Cepoiu-Martin et al. revealed that increasing age (1-year increments) is significantly associated with a higher risk of NH admission [14]. Additionally, a higher risk of NH admission has been reported for unmarried (widowed, divorced or single) people with dementia compared to married persons [13, 14]. People living alone have a higher risk of NH admission compared to those living with a spouse or a caregiver [12, 13]. Severity of cognitive impairment (e.g. as measured by the Mini-Mental Status Examination or Clinical Dementia Rating Scale) is a significant predictor of increased risk of NH admission [13–16]. Functional impairments and basic activity of daily living dependencies significantly increase the risk of NH admission [13–16]. Furthermore, occurrence of neuropsychiatric symptoms, such as aggression, anxiety, depression and psychosis, have been reported to increase the risk of NH admission [13–16]. Poorer physical health and severity of immobility have also been associated with NH admission [16]. Caregiver characteristics associated with NH admission have been identified in several studies. Having a female caregiver may reduce the risk of NH admission [14], whereas the caregiver being an adult child is associated with earlier institutionalisation of the person with dementia, compared to spousal caregivers [13]. Poorer physical health, heavier burden [12–16] and depressive symptoms of the caregivers have also been associated with a higher risk of NH admission [13, 14].

Previous research studying the impact of various services, such as a day care programme, support programmes and in-home help, has shown inconsistent results regarding the postponement of NH admission. An increased risk of NH admission has been reported for people who attend a day care programme for more days and for those who use community health services more often [12, 17, 18]. In contrast, other studies have demonstrated that receiving day care services and the intensity of community home care services increase the probability of remaining at home and not being admitted to residential care [19]. Furthermore, utilisation of in-home help earlier in the course of dementia has been reported to delay institutionalisation [20]. A meta-analysis by Spijker et al. [21] explored the effectiveness of nonpharmacological interventions in delaying institutionalisation of people with dementia. In total, 13 studies of support programmes were identified with follow-up periods ranging from three to 102 months. The participants were all community-dwelling, but five of the interventions took place in outpatient settings, like day care, university or mental health services. The programmes were all multicomponent, offering individualised interventions to meet the needs of the patients and their caregivers. The meta-analysis showed that patients included in the interventions were significantly less likely to be institutionalised than patients in control groups and, furthermore, the time to institutionalisation significantly increased. The analysis of the intervention characteristics showed that actively involving family caregivers in decisions about treatments distinguishes effective from ineffective programmes [21].

The aim and content of day care as an intervention designed for people with dementia is not standardised, and, hence, the evaluation of its effects is a challenge [4, 22]. In most previous studies exploring the effect of day care programmes on NH admission, the intervention has been described as adult day care with no further information about the content of the programmes or their suitability for people with dementia.

At present, there is only limited knowledge concerning the effects of day care programmes designed for people with dementia. A review conducted by the Norwegian Knowledge Centre for the Health Service (NOKC) in 2011 [23] identified a mere eight published studies of moderate to poor quality. At the societal level, two of these studies reported lower costs for the day care group, due to reduced admittance to acute hospitals [24] or nursing homes [25]. However, cost effectiveness could not be confirmed [26]. An updated review conducted by NOKC in 2014 was not able to identify any further research in this field [27].

In a review, Luppa et al. [13] concluded that there is a need for development and application of interventions that can maintain people with dementia to live in their own homes for a longer period to assure timely institutionalisation.

The recent systematic review and meta-analysis of predictors of long-term placement in persons with dementia by Cepoiu-Martin et al. concluded that important information about several individual and caregiver predictors of NH admission are available. However, other factors, such as day care services, show inconsistent effects [14]. Thus, additional research to gain valid knowledge about whether day care programmes designed for people with dementia can postpone admittance to nursing homes is needed.

## Methods

### Study aim, design and setting

The main objective of this study is to explore the impact of a day care centre programme designed for people with dementia on NH admission. This is a quasi-experimental study comparing a group of people with dementia receiving a day care centre programme designed for people with dementia (day care group, DG) with a group of people with dementia not receiving day care, as they did not live in a municipality with such a service (comparison group, CG). Both groups were followed up after 24 months.

### Participants

The following inclusion and exclusion criteria were employed: participants should be 65 years or older, have a dementia diagnosis according to the ICD-10 criteria, a Mini-Mental State Examination (MMSE) score of > 15 and the capacity to give informed consent based on the judgement of a professional caregiver. The inclusion of people with MMSE scores > 15 was chosen as an additional criterion to ensure the probability for the participants’ capacity to give informed consent. Additionally, a family caregiver of each participant should be willing to participate and give his or her informed consent to participate. Both the patient and the family caregiver had to give consent. Patients in the day care group should have attended the centre for at least 4 weeks and should not have been in the programme for longer than 12 months. Furthermore, they had to attend the centre at least twice a week to be included. Participants who had applied for a permanent nursing home placement or were suffering from a serious co-morbid physical disorder with a life expectancy of less than 6 months were excluded. The inclusion of participants was made between December 2013 and July 2015 and follow-up was made 24 months after inclusion.

### Power calculation and recruitment

**Fig. 1:**
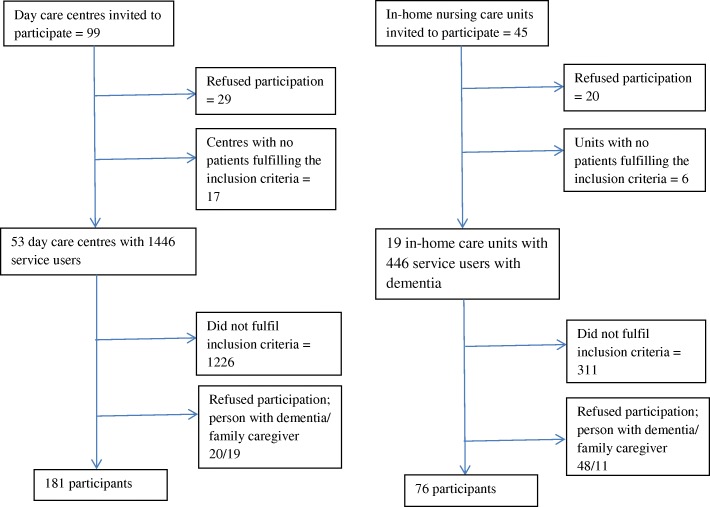
Recruitment of participants

The primary outcome in the study was the proportion of patients admitted to nursing home care by the 24-month follow-up. In accordance with the results of Engedal [24] and Taranrød [28], we expected that 47% of the patients receiving day centre services would be admitted to nursing home care within 24 months, compared to 62% of the patients in the CG. To demonstrate a significant difference between the groups with 80% power and a significance level of 5% with a two-way χ^2^-test, a sample size of 172 patients in each group was required. We planned to include 200 patient-family caregiver dyads in each group, allowing for a 15% drop-out rate. Thus, the study sample was planned to consist of 400 patients with dementia living at home and their primary family caregivers. Participants in the DG were recruited from day care centres designed for people with dementia, and patients in the CG were recruited from local authority dementia teams and in-home care service offices in municipalities without day care centre programmes for this patient group. The participants were recruited from various municipalities spread across Norway, including small and large municipalities in both groups. Both participants in DG and CG received in-home care services based on the assessment of individual needs made by the municipalities. The recruitment and inclusion process is illustrated in Fig. 1. A total of 99 day care centres and 45 in-home nursing care units were initially invited to recruit participants for the study, with 53 day care centres and 19 in-home units taking part in the final recruitment process. In DG, 39 (18%) of the 220 participant dyads that fulfilled the inclusion criteria refused participation (20 patients and 19 caregivers) In CG, 59 (44%) of the 135 invited participant dyads refused participation (48 patients and 11 caregivers). The sample at baseline comprised 181 participants in the DG and 76 participants in the CG.

### Intervention and comparison condition

The intervention consisted of a stay in a day care centre designed for people with dementia for at least 2 days a week. Any type of day care centre with a programme designed for people with dementia was eligible for inclusion in the study. To receive access to day care designed for people with dementia in Norway, the attendee should be diagnosed with dementia. The day care services could take place at an institution, such as a nursing home unit, meeting centre, farm or another suitable location. Central aims of day care should be to offer meaningful activities to the attendees and respite for family caregivers [29]. The comparison condition was “care as usual”.

### Data collection

A group of 13 trained assessors, consisting of nurses, occupational therapists and a psychologist, all experienced in dementia care and with no connection to the sample organisations, collected the data. Before data collection, they received information about the study and 1 day of training in the use of the assessment tools.

Data were collected at baseline and after 24 months. Information about NH admission was recorded as yes (1) or no (0) at the 24-month follow-up. Demographic data of patients and family caregivers such as gender, age, marital status, living condition and educational level, were collected. To diagnose dementia, a standardised and structured assessment of symptoms, debut, type and course was used at baseline. Two experienced psychiatrists independently diagnosed the participants according to the ICD-10 criteria for research using all the available information from the data collection. Discrepancies in diagnoses were settled in a consensus meeting. At baseline and follow-up, the following assessments were made.

#### Clinical Dementia Rating scale

The severity of dementia was assessed by the Clinical Dementia Rating scale (CDR) [30], a six-item assessment scale. Using an algorithm giving precedence to memory, the severity of dementia is staged as (0) none, (0.5) possible, (1) mild, (2) moderate or (3) severe dementia. The reliability of the Norwegian version of CDR has been evaluated to be adequate [31].

#### Mini-Mental State Examination

The Mini-Mental State Examination (MMSE) [32] was used to assess cognitive functioning. The scale consists of 20 items, with a possible sum score between 0 and 30. A higher score indicates better cognitive function. The MMSE has been evaluated to be a suitable tool for the detection of cognitive impairment in Norwegian patients of old age [33].

#### Activities of daily living

Functioning in activities of daily living (ADL) was assessed by the Physical Self-Maintenance Scale (PSMS) and the Instrumental ADL scale (IADL) [34]. The PSMS scale measures basic activities and has six items rated on a five-point scale from one (maintain the activity) to five (do not maintain the activity at all/need full assistance). The minimum score is six and the maximum is 30. The IADL scale assesses the instrumental activities of daily living and has eight items. The scoring system is similar to that of the PSMS, but the maximum score on each item varies between three and five, which gives a minimum score of eight and a maximum of 31. For both scales, a higher score indicates poorer functioning and a greater need for assistance. The scales have utility in widely diverse settings with a range of population groups [34] and have been used in several previous Norwegian studies of home-dwelling people with dementia [11, 35] .

#### The Montgomery-Asberg Depression Rating Scale

Depressive symptoms were assessed by the Montgomery-Asberg Depression Rating Scale (MADRS) [36], which has been validated and found reliable for use among people with dementia [37]. MADRS was used in an interview with the participants. The scale consists of 10 items that can be scored from zero to six, giving a minimum score of zero and a maximum score of 60, with a higher score indicating more severe depressive symptoms.

#### The Neuropsychiatric Inventory Questionnaire

The Neuropsychiatric Inventory Questionnaire (NPI) [38] was used to evaluate neuropsychiatric symptoms. The NPI has been validated and found reliable [39]. The questionnaire was used in an interview with the family caregiver to assess the presence of NPS. If present, the severity of each of the 12 symptoms was assessed on a scale from one to three, giving an item score ranging from zero to three and a sum score ranging from zero to 36. [39]. Based on previous principal component analyses made among home-dwelling people with dementia, the sub-scores NPI-psychosis (delusions and hallucinations), NPI-affective (depression, anxiety and apathy) and NPI-hyperactivity (agitation, disinhibition, euphoria, irritability and aberrant motor behaviour) were used [11, 40]. Additionally, NPI-sleep was included in the analysis as a single item.

#### The Anosognosia Rating Scale

The Anosognosia Rating Scale (REED) [41] was used to rate the patients’ degree of awareness of memory loss. REED consists of a four-point scale (1– full awareness, 2 – shallow awareness, 3 – no awareness and 4 – denies impairment); the scorings are based on an interview, which is often combined with cognitive testing of the patient. This scale has high inter-rater reliability [41], and it correlates well with the discrepancy score between the informants’ and patients’ reports [42].

#### Relatives’ Stress Scale

Caregivers’ level of stress related to caregiver burden was examined using the Relatives’ Stress Scale (RSS) [43]. The RSS consists of 15 questions with five alternative answers with a scoring range from zero (never) to four (very often/always), giving a minimum score of zero and a maximum score of 60, with a higher score indicating more severe stress. The scale has been evaluated to be a useful tool to identify various aspects of carer burden in dementia [44].

Additionally, at the family caregiver level, depressive symptoms were also assessed using the MADRS.

### Statistical analysis

Missing values on the different instruments were imputed at the item level. The imputation was performed only for cases with at least 50% of items on the scale available. The missing values (all 12 for MADRS patient, 13 for MADRS family caregiver,13 for RSS, and 37 of 51 for IADL) were imputed by random numbers drawn from an empirical distribution generated for each item of interest.

Demographic and clinical characteristics were presented as means and standard deviations (SD), or frequencies and percentages as appropriate. The differences between the DG and the CG in permanent admission to nursing home after 24 months were assessed using the χ^2^-test. A logistic regression model was further applied. The main predictor in the model was participant group (DG or CG). Other predictors were predefined a priori to analyses and included age, gender, level of education, living alone, MMSE sum, MADRS sum for patient, REED sum, NPI-psychosis, NPI-affective, NPI-hyperactivity, NPI-sleep, IADL sum, PSMS sum, MADRS sum family caregivers and RRS, all measured at baseline. First, unadjusted (bivariate) regression models were estimated for each predictor. Next, an adjusted (multiple) model containing all predictors and interaction terms between participant group and each predictor was estimated. A significant interaction implies that there are differences in permanent admission to nursing homes between patients in the DG and patients in the CG with respect to association. The adjusted model was further reduced by Akaike’s Information Criterion (AIC), where a smaller value implies a better model. Adjustment for intra-centre correlations was considered but omitted as correlations were negligible. The results were presented as regression coefficients and standard errors (SE) for variables included in the interactions and as odds ratio (OR) with a 95% confidence interval (CI) otherwise.

The statistical analyses were performed in SPSS v 25 and SAS v 9.4. Results with *p*-values below 0.05 were considered statistically significant.

### Ethical consideration

The project was approved by the Regional Committee in Ethics in Medical Research in South-East Norway (2013/1020). After being given written and oral information, the patients and the family caregivers were asked to give written informed consent. Only patients with the capacity to give consent were included.

## Results

The baseline descriptive data of the participants are presented in Table 1. At baseline, 257 participants were included, 181 (70.4%) in the DG and 76 (29.6%) in the CG. The mean age of the patients was 81.5, SD 6.4. There were 168 female participants (65.4%), and 135 participants (52.5%) lived alone. The mean MMSE score was 20.4, SD 3.5, and 88 of the participants (34.2%) had full awareness of their memory loss according to the REED scale. The mean age of the family caregivers was 62.0, SD 13.1, and 177 (68.90%) were women. A total of 94 of the participating caregivers (36.6%) lived with the patient, and these were mainly spouses. In total, there were 87 (33.9%) spousal caregivers.

**Table 1:** Baseline characteristics patients and family caregivers

Patient characteristics	Day care group (*N* = 181)	Comparison group (*N* = 76)	All (*N* = 257)
Age, mean (SD)	81.1(6.5)	82.4 (6.0)	81.5 (6.4)
Female gender, n (%)	110 (60.8)	58 (76.3)	168 (65.4)
Living alone, n (%)	92 (50.8)	43 (56.6)	135 (52.5)
Education after primary school, n (%)	87 (48.1)	25 (32.9)	112 (43.6)
Full awareness (REED), n (%)	62 (34.3)	22 (28.9)	84 (32.7)
MMSE score, mean (SD)	20.5 (3.5)	20.3 (3.6)	20.4 (3.5)
MADRS score, mean (SD)	4.7 (4.8)	5.9 (5.4)	5.1 (5.0)
PSMS score, mean (SD)	9.5 (3.2)	9.3 (3.1)	9.5 (3.2)
IADL score, mean (SD)	22.6 (5.1)	20.4 (5.6)	21.9 (5.3)
NPI-psychosis sub score, mean (SD)	0.9 (1.4)	0.6 (1.2)	0.8 (1.4)
NPI-affective sub score, mean (SD)	2.3 (2.1)	2.0 (1.9)	2.2 (2.0)
NPI-hyperactivity sub score, mean (SD)	2.0 (2.2)	1.4 (2.1)	1.8 (2.2)
NPI-sleep item score, mean (SD)	0.5 (0.8)	0.4 (0.8)	0.5 (0.8)
Dementia diagnosis, n (%)			
• Alzheimer’s disease (AD)	140 (77.3)	62 (81.6)	202 (78.6)
• Vascular dementia (VD)	20 (11.0)	2 (2.6)	22 (8.6)
• Mixed AD/VD	8 (4.4)	6 (7.9)	14 (5.4)
• Lewy body dementia/Parkinson’s disease with dementia	11 (6.1)	2 (2.6)	13 (5.1)
• Frontotemporal dementia	2 (1.1)	0	2 (0.8)
• Other dementias	0	4 (5.2)	4 (1.6)
Severity of dementia CDR, n (%)			
• CDR 0.5 – possible dementia	22 (12.2)	20 (26.3)	42 (16.3)
• CDR 1 – mild dementia	117 (64.6)	48 (63.2)	165 (64.2)
• CDR 2 – moderate dementia	41 (22.7)	8 (10.5)	49 (19.1)
• CDR 3 – severe dementia	1 (0.6)	0	1 (0.4)
Family caregiver characteristics			
Age, mean (SD)	62.4 (13.5)	61.1 (12.2)	62.0 (13.1)
Female gender, n (%)	125 (69.1)	52 (68.4)	177 (68.9)
Live together with the patient, n (%)	76 (42.0)	18 (23.7)	94 (36.6)
Relationship to the patient			
• Spouse	69 (38.1)	18 (23.7)	87 (33.8)
• Child/Child-in-law	106 (58.6)	54 (71.1)	160 (62.3)
• Other	6 (3.3)	4 (5.3)	9 (3.5)
MADRS score, mean (SD)	3.6 (4.3)	3.7 (4.9)	3.7 (4.5)
RSS score, mean (SD)	19.1 (11.1)	14.1 (4.9)	17.7 (11.0)

*REED* The Anosognosia Rating Scale, *MMSE* Mini Mental State Examination, *MADRS* Montgomery-Asberg Depression Rating Scale, *PSMS* Physical Self-Maintenance Scale, *IADL* Instrumental Activities of Daily Living, *NPI* Neuropsychiatric Inventory Questionnaire, *CDR* Clinical Dementia Rating scale, *RSS* Relatives’ Stress Scale

The drop-out rate and the number of participants admitted to nursing homes in the study sample from baseline to follow-up at 24 months are presented in Table 2. A total of 63 participants (24.5%) completed the follow-up assessment at 24 months. Altogether, 128 (49.8%) dropped out due to NH admission (93 (51.4%) in DG and 35 (46.1%) in CG), 20 (7.8%) died and 46 (17.9%) dropped out due to withdrawal or other reasons. The participants who dropped out had a significantly lower MMSE mean score at baseline 20.0 (SD 3.5) compared to the participants who completed follow-up, 21.7 (SD 3.1), *p* = 0.001. Additionally, the drop-outs at follow-up had a significantly higher mean IADL score of 22.4 (SD 5.1) and PSMS 9.8 (SD 3.2) compared to the participants who completed follow-up, IADL 20.4 (SD 5.6) *p* = 0.009 and PSMS 8.5 (SD 2.6) *p* = 0.005. Furthermore, the drop-outs had a significantly higher NPI-psychosis score of 1.9 (SD 1.5) versus 0.5 (SD 0.9) *p* = 0.018 in the participants that completed the follow-up.

**Table 2:** The drop-out rate and the number of participants admitted to nursing homes from baseline to follow-up after 24 months in day care group and comparison group

	Day care group n (% compared to baseline)	Comparison group n (% compared to baseline)	Both groups n (% compared to baseline)
Baseline, participants	181 (70.4)	76 (29.6)	257 (100)
After 24 months, participants	47 (26.0)	16 (21.1)	63 (24.5)
Nursing home admission	93 (51.4)	35 (46.1)	128 (49.8)
Dead	15 (8.3)	5 (6.6)	20 (7.8)
Dropped out:	26 (14.4)	20 (26.3)	46 (17.9)
• Withdrew	15	13	38
• Other reasons	11	7	18

No differences in NH admission were found between DG and CG (*p* = 0.743). The results of the logistic regression model for NH admission after 24 months are presented in Table 3.

**Table 3:** Predictors of nursing home admission after 24 months. Results of logistic regression model with covariates measured at baseline, unadjusted and adjusted AIC-reduced models (*N* = 194)

Variables	Unadjusted model		Adjusted AIC-reduced model^a^	
	OR (95% CI)	*p*-value^2^	OR (95% CI) /Regression coefficient (SE)	*p*-value^2^
Group (comparison group – ref.)	0.92 (0.47; 1.80)	0.814	19.78 (7.06)^a^	**0.006**
Age	1.02 (0.98; 1.07)	0.348	0.20 (0.07)^a^	**0.006**
Gender (women – ref.)	0.60 (0.33; 1.10)	0.104	0.46 (0.20; 1.06)	0.071
Education (education after primary school – ref.)	0.59 (0.33; 1.06)	0.079		
Living alone	1.56 (0.87; 2.79)	0.138	1.65 (1.00)^a^	0.101
MMSE sum	0.83 (0.75; 0.91)	**< 0.001**	0.80 (0.72; 0.90)	**< 0.001**
MADRS sum patient	1.02 (0.96; 1.07)	0.596		
REED sum	0.84 (0.52; 1.36)	0.486	0.65 (0.37; 1.14)	0.135
NPI-psychosis	1.35 (1.06; 1.73)	**0.016**		
NPI-affective	1.08 (0.93; 1.25)	0.345	0.48 (0.26)^a^	0.061
NPI-hyperactivity	1.07 (0.93; 1.23)	0.334	1.16 (0.96; 1.39)	0.120
NPI-sleep	0.91 (0.63; 1.31)	0.605	−1.12 (0.56)^a^	**0.048**
IADL sum	1.05 (0.99; 1.11)	0.114		
PSMS sum	1.09 (0.99; 1.21)	0.075	0.29 (0.15)^a^	**0.049**
RSS sum, caregivers	1.01 (0.99; 1.04)	0.370		
MADRS sum, caregivers	0.99 (0.93; 1.06)	0.874		
Group x age			−0.19 (0.08)	**0.013**
Group x living alone			−1.79 (1.07)	0.095
Group x NPI-affective			−0.48 (0.27)	0.079
Group x NPI-sleep			1.15 (0.62)	0.065
Group x PSMS-sum			−0.23 (0.16)	0.142

*MMSE* Mini Mental State Examination, *MADRS* Montgomery-Asberg Depression Rating Scale, *REED* The Anosognosia Rating Scale, *NPI* Neuropsychiatric Inventory Questionnaire, *IADL* Instrumental Activities of Daily Living, *PSMS* Physical Self-Maintenance Scale, *RSS* Relatives’ Stress Scale

^2^*p*-value < 0.05 is considered significant

^a^Coefficients and SE are presented instead of OR and 95% CI for variables included into the interaction terms in the adjusted model, since the OR for such variables is not interpretable

*P*-values in boldface are <0.05 and considered significant

Participant group (DG or CG) was not found to be a significant predictor of NH admission in the unadjusted model (Table 3). Odds of nursing home admission were higher for those with a lower MMSE score (*p* < 0.001), indicating more severe cognitive impairment, and a higher NPI-psychosis score (*p* = 0.016), indicating the occurrence of psychotic symptoms of hallucinations and delusions.

In the adjusted AIC-reduced model (Table 3), lower MMSE scores were still associated with higher odds of nursing home admission (*p* < 0.001). Participant group (DG or CG) was associated with NH admission through several interactions. The odds of NH admission were significantly higher for DG than CG for all ages up to 82 years. The odds of NH admission increased significantly with increasing age in CG (*p* = 0.006) but remained stable in DG.

The odds of NH admission were significantly higher in the DG group compared to CG among both those living alone (*p* = 0.005) and those living with somebody (*p* = 0.008). The odds of NH admission were significantly higher in the DG compared to the CG for all NPI-affective values. The odds of NH admission increased non-significantly with increasing NPI-affective sub-score (*p* = 0.056) in CG but remained stable in DG.

The odds of NH admission were significantly higher for DG than CG for all NPI-sleep score values. However, increase in NPI-sleep score values (indication of more sleeping symptoms) reduced the odds of NH admission in CG (*p* = 0.047) but remained stable in DG.

The odds of NH admission were significantly higher in DG than CG for all PSMS scores. The odds of NH admission increased significantly with increasing PSMS score values in CG (*p* = 0.048) but remained stable in DG.

## Discussion

The main objective of the study was to explore the impact of day care designed for people with dementia on NH admission. The results from the adjusted logistic regression model revealed that participant group (DG or CG) was associated with NH admission through the interactions with age, living conditions, affective symptoms, sleep symptoms and practical functioning, showing higher odds of NH admission in DG compared to CG.

These findings are important to illuminate the expectations of the benefits from day care services and determine the potential for day care to postpone the need for NH admission. The findings from the current study, revealing an increased risk for NH admission in the DG group compared to the control, are comparable with previous studies. For example, a study by McCann et al. revealed that the risk of NH admission increased with the number of days of adult day care attendance for people with dementia. Additionally, heavier caregiver burden increased the risk [17]. Wattmo et al. reported that more days in adult day care at baseline or a substantial increase in the use of day care during 36 months of follow-up predicted earlier admission to nursing homes among participants with Alzheimer’s disease [18]. Furthermore, our findings are in line with previous reviews exploring the predictors of NH admission, concluding that demographic characteristics such as advanced age and living alone, and clinical characteristics including more cognitive and ADL impairment and the occurrence of neuropsychiatric symptoms, increase the probability of NH admission [13–16]. The participants in DG were more dependent in activities of daily living as measured by IADL and PSMS at baseline compared to CG, and there was a higher proportion of participants with moderate/severe dementia in DG versus CG. Additionally, there is a possibility that the results are confounded by indication as the DG showed higher scores on NPI at baseline compared to CG, a possible indication of a more complicated and demanding situation for caregivers to handle. Based on the staff’s ongoing evaluation of the development of the attendees’ impairment and needs, they might encourage and support the caregivers in making the difficult decision to apply for NH admission. Thus, there could be differences between users and non-users of day care that can add information to the increased odds of NH admission.

Previous research has revealed that caregiver characteristics such as higher scores of caregiver burden and higher occurrence of physical health problems increase the probability of NH admission [14–16]. In the current study, no caregiver characteristics were significantly associated with NH admission.

An additional potential explanation for the increased odds of NH admission in the DG compared to the CG is that day care services are introduced in the moderate to late stage of dementia, when stress and burden have become too high for caregivers to master. As such, the service will serve more as a transitional period to residential care than as a respite to postpone the need for NH admission [18]. As stated in a recent review by Du Preez et al. (2018), intervention early in the caregiving process can offset caregiver stress through utilisation of adult day service support, respite and education [45]. Qualitative research, conducted as part of the current project, adds important information about the experience of day care from the perspective of family caregivers. The family caregivers experience respite and reassurance from the service [46, 47]. Additionally, the potential for day care to increase family caregivers’ motivation to care and increase their feeling of mastery has been demonstrated [48]. Based on these positive experiences of day care, the potential for the services to postpone the need for NH admission could be presumed. However, despite the promising findings from the interviews, the day care services did not delay NH admission. A potential explanation for this unexpected result could be that when the family caregivers experienced respite and support from professional staff, they may have recognised their need for further support and relief of burden. This interpretation can be supported by higher scores of caregivers’ burden (RSS) at baseline in DG compared to CG. If family caregivers experience reassurance due to high quality care for their next-of-kin, they might be more positive in accepting the need for NH admission based on increased trust in the health and care services.

The content and organisation of the day care should also be considered when interpreting the results. Day care services aim to increase participation in a range of activities and enhance the quality of life and well-being of the attendees, and the benefits of the service have been demonstrated [49–51]. A review by Du Preez et al. highlighted that family caregivers often have limited contact with the day care services, and this lack of contact coupled with the inability of the day care services to offer person-centred activities contribute to non-utilisation of the services. However, when day care services actively invite caregivers to collaborate in the day care programme and provide caregiver education, counselling and support, institutionalisation is delayed [45]. Interviews with caregivers in the current project demonstrated the need for more flexibility in the number of opening days and opening hours. Additionally, they expressed their need for more individualised care tailored to both their own needs and the needs of the person with dementia. In the caregivers’ opinion, the health and functional level of the day care attendee, especially with relation to personal hygiene, was important in postponing NH admission. As experienced by the caregivers, their daily support supplemented by professional home care was crucial to postpone NH admission in addition to the support from day care [47].

### Strengths and limitations

A strength of this study is the inclusion of participants from a large number of day care centres of various types, based in municipalities of different sizes spread all over Norway. The follow-up period was 2 years, and standardised test and evaluation instruments that have been widely used in previous research on this patient group were used in the assessments. The assessment tools have been tested for reliability and validity in Norwegian studies, strengthening the reliability and validity of the present findings.

The design of the study has some limitations. A randomised controlled trial (RCT) design, considered to be the gold standard when studying effects of interventions, is preferable and would have increased the possibility for generalisation. However, the waiting list period for day care centre admittance is short in Norway. We therefore found it unethical to withhold patients from this service during a 24-month observation period, and, as a substitute for an RCT, we chose to carry out a quasi-experimental controlled trial. Eligible patients in the local authorities (identified by day care staff), dementia teams and in-home care offices were asked to participate. However, there might have been differences between those who accepted the recruitment proposal and those who declined.

Additionally, the results need to be considered in accordance with the limitation of the high drop-out rate and the lack of power we were able to obtain. The inclusion of participants in the comparison group was a challenge. This could have different possible reasons. The provision of day care services has been a governmental priority for several years [7, 8], and many municipalities have day care designed for people with dementia that limits the availability of CG participants. Furthermore, to obtain access to participants in CG, there was a need for assistance from home-care nurses to identify potential participants who fulfilled the inclusion criteria, provide written information and be prepared to add oral information to receive the participants’ consent to be contacted by the research group. Feedback from some of the home-care service units that agreed to recruit participants indicated that the nurses found this task complicated and time consuming and, hence, it was given lower priority. Additionally, the drop-out rate was higher than estimated in the power calculation. Thus, the results cannot be considered generalisable to the population of people with dementia with an MMSE score > 15. However, the direction of the results might indicate no potential positive effect of day care designed for people with dementia on NH admission.

## Conclusion

The study reveals no evidence to confirm the hypothesis that day care services designed for people with dementia have the potential to delay the need for NH admission. These findings confirm the knowledge presented in previous research but need to be considered in accordance with the limitation of the high drop-out rate and the inadequate power in the study. The need for NH admission is based on a complex mix of personal and functional characteristics in the person with dementia and the needs of his or her family caregivers. The influence of the design and flexibility of the day care programme, the combination of day care and in-home nursing services and the role of caregiver support and involvement in designing the day care programme should be further investigated.

## Abbreviations

AD: Alzheimer’s disease
ADL: Activities of daily living
AIC: Akaike’s Information Criterion
CDR: Clinical Dementia Rating scale
CG: Control group
CI: Confidence interval
DG: Day care group
IADL: Instrumental Activities of Daily Living
MADRS: Montgomery-Asberg Depression Rating Scale
MMSE: Mini Mental State Examination
NH: Nursing home
NOKC: Norwegian Knowledge Centre for the Health Services
NPI: Neuropsychiatric Inventory Questionnaire
OR: Odds ratio
PSMS: Physical Self-Maintenance Scale
RCT: Randomised controlled trial
REED: The Anosognosia Rating Scale
RSS: Relatives’ Stress Scale
SAS: Statistical analysis system
SD: Standard derivation
SE: Standard error
SPSS: Statistical Package for the Social Sciences
VD: Vascular Dementia
